# Novel Opipramol-Baclofen Combination Alleviates Depression and Craving and Facilitates Recovery From Substance Use Disorder—An Animal Model and a Human Study

**DOI:** 10.3389/fnbeh.2021.788708

**Published:** 2021-12-23

**Authors:** Tzofnat Bareli, Hadas Levi Ahdoot, Hila Ben Moshe, Royi Barnea, Gal Warhaftig, Iris Gispan, Rachel Maayan, Paola Rosca, Abraham Weizman, Gal Yadid

**Affiliations:** ^1^Faculty of Life Sciences, Leslie and Gonda (Goldschmied) Multidisciplinary Brain Research Center, Bar Ilan University, Ramat Gan, Israel; ^2^The Laboratory of Molecular Psychiatry, Felsenstein Medical Research Center, Petah Tikva, Israel; ^3^Faculty of Medicine, Tel Aviv University, Tel Aviv, Israel; ^4^Department for the Treatment of Substance Abuse and Mental Health Services, Israeli Ministry of Health, Jerusalem, Israel; ^5^Faculty of Medicine, The Hebrew University of Jerusalem, Jerusalem, Israel; ^6^Research Unit, Geha Mental Health Center, Petah Tikva, Israel

**Keywords:** stress, substance-induced depressive disorder, addiction, treatment, therapeutic center, baclofen, opipramol

## Abstract

Substance use disorders (SUDs) are associated with depression and anxiety, with the latter being one of the major factors in substance-seeking and relapse. Due to dose-dependent sedative side effects there is limited efficacy of baclofen treatment for SUDs. Here we suggest the use of a novel combination of opipramol and baclofen (O/B) which is known to attenuate anxiety and depression, for the facilitation of recovery from SUDs. Since both opipramol and baclofen have a common downstream signal transduction, their individual doses could be reduced while still maintaining the benefits of the combination. We tested the O/B combination in both animals and patients. Rats treated with O/B showed significant attenuation in craving behavior and in relapse rate during withdrawal from cocaine. In a double-blind, placebo-controlled pilot study, conducted in a residential detoxification center, 14 males and 3 females, aged 28–60 years were assigned to a study (*n* = 6) and a placebo (*n* = 11) group (placebo group: 40 ± 10.5 years; O/B group 40 ± 10.8 years). The participants completed scales measuring depression, anxiety and craving symptoms and provided saliva samples for stress hormone examination [cortisol and dehydroepiandrosterone-sulfate (DHEA-S)]. Participants with polysubstance use disorder (PsUD) treated with O/B showed a reduction in cravings and depression and an increase in DHEA-S and in the DHEA-S/cortisol ratio. Our findings indicate a beneficial effect of O/B treatment. This study suggests a novel candidate for pharmacological treatment of patients with SUD and comorbid mood/anxiety disorders that may facilitate their rehabilitation.

## Introduction

Substance use disorder (SUD) affects every aspect of a person’s life, as well as their social environment. It may destroy relationships, lead to unemployment, poor health, and mortality due to overdose. The Diagnostic and Statistical Manual of Mental Disorders (DSM-5) substituted in 2013 the previous dichotomous definition between substance abuse and dependence, with the term SUD relating to it as a continuum. Substance-induced depressive disorder (SIDD) refers to depression induced by substance use (Magidson et al., 2013; Conner et al., 2014; Tolliver and Anton, 2015; Tirado-Muñoz et al., 2018).

Opipramol is a well-tolerated anti-anxiety and antidepressant agent (Möller et al., 2001). Clinical trials with opipramol showed it to be effective in anxiety associated with somatic complaints (Möller et al., 2001). Opipramol is a σ-1 receptor agonist. This receptor is defined as a chaperone and can modulate the activity of several ion channels thus affecting neuronal excitability and synaptic activity (Su et al., 2016; Yang et al., 2019) and may be involved in cocaine addiction (Ferris et al., 1991; Katz et al., 2016). Recently, we showed significant reduction of cocaine-primed reinstatement in 75% of the opipramol-treated group as well as demonstrating the involvement of σ-1 receptor in a rat model of cocaine self-administration (SA) (Bareli et al., 2021). In addition, we found that those rats which responded to opipramol (Responders = Rs) exhibited significantly less Ras-related C3 botulinum toxin substrate 1 (Rac1) mRNA expression in the nucleus accumbens (NAc), compared with opipramol non-responder (NR) rats. Hence, we concluded that Rac1 differentiated Rs from NRs (Bareli et al., 2021). A study by Tsai et al. (2012) demonstrated that σ-1 receptor knock-down in the hippocampus is associated with a parallel downregulation of gamma-aminobutyric acid B receptor 1 (GABA_*B*_,Gabbr1) and Rac1.

Baclofen is a potent, selective Gabbr1 agonist (Bowery et al., 1980, 1981; Davidoff, 1985) and is used in clinical practice as a muscle relaxant in patients suffering from multiple sclerosis and spinal cord diseases (Kuroiwa et al., 2009; Woolf and Baum, 2017; Lake and Shah, 2019; Chisari et al., 2020). Additionally, a variety of studies show that the GABA_*B*_ receptor is involved in SUDs (Roberts and Andrews, 1997; Di Ciano and Everitt, 2003; Agabio et al., 2013; Phillips and Reed, 2014), including ethanol (EtOH) consumption. In mice, the GABA_*B*_ receptor agonist baclofen decreases EtOH intake (Rabat et al., 2019) and attenuates social-interaction deficits when given before ethanol exposure or anxiety during abstinence (Knapp et al., 2007). However, side effects, such as severe sedation, dizziness, and confusion, were observed as a result of high-doses (de Beaurepaire et al., 2019). In a study on people with severe cocaine dependence, the benefit of baclofen as a sole treatment was inconclusive compared to placebo (Kahn et al., 2009).

Chronic anxiety enhances the susceptibility to SUDs (Sinha and Jastreboff, 2013). In a previous study the authors showed that relieving anxiety in rats with the neurosteroid dehydroepiandrosterone (DHEA), a σ-1 receptor agonist, is associated with a reduction in cocaine SA and cocaine-seeking during abstinence (Yadid et al., 2012). The DHEA sulfate ester (DHEA-S) and DHEA, are the most abundant steroid hormones in the body. In humans with SUD, the levels of DHEA and DHEA-S are altered (Buydens-Branchey et al., 2002). Notably, DHEA-S levels were significantly lower in patients with SUD who relapsed, compared to patients that did not relapse (Shoptaw et al., 2004).

The present study suggests attenuating anxiety and depression during withdrawal from SUD by using an opipramol and baclofen (O/B) combination. Baclofen at low doses is known to be well-tolerated and safe. The downstream signal transduction which is common to both opipramol and baclofen allows reduction of the baclofen dose, thus diminishing the sedative side effects. We postulated that the O/B combination mediated by σ-1 receptor/Gabbr1 interaction and possibly by other proteins like Rac1, could provide a new strategy for decreasing drug cravings. The current study evaluated the efficacy of O/B treatment for SUD as well as comorbid anxiety/depressive symptoms. In the preclinical phase the efficacy was examined in a rat model of cocaine use disorder. The second clinical phase consisted of a double-blind, placebo-controlled pilot study conducted in a detoxification residential center including patients with polysubstance use disorder (PsUD). The diagnosis of PsUD refers, in our study, to a situation in which an individual uses at least two different classes of substances repeatedly (excluding caffeine or nicotine) during a 12-months period, but no single substance predominates (Martinotti et al., 2009).

In the current study, the authors aim to show that the O/B combination can alleviate anxiety and depression associated with SUD thus facilitating rehabilitation.

## Study 1—Animal Model

### Materials and Methods

#### Ethics

All experimental procedures were approved by the Animal Care and Use Committee of Bar Ilan University, Ramat Gan, and were performed in accordance with the National Institutes of Health guidelines for animal experiments.

#### Animals

Male Sprague-Dawley rats (250–280 g) were maintained on a reverse 12–12-h dark-light cycle with free access to food and water.

#### Intravenous Catheterization

Rats were anesthetized with ketamine hydrochloride (100 mg/kg, i.p.)/xylazine (10 mg/kg, i.p.) and then implanted with intravenous silastic catheters (Dow Corning, Midland, MI, United States) into the right jugular vein. The catheter was secured to the vein with silk sutures and passed subcutaneously to the top of the skull, where it exited into a connector (a modified 22-gauge cannula; Plastics One, Roanoke, VA, United States) mounted to the skull with MX-80 screws (Small Parts, Inc., Miami Lakes, FL, United States) and dental cement (Yates & Birds, Chicago, IL, United States).

#### Cocaine Self-Administration Training

Rats were trained to self-administer cocaine (between 10 and 16 days depending on the rate of addiction in every experiment) under a fixed ratio (FR)-1 schedule of reinforcement. Five days after catheterization, rats were transferred to operant conditioning chambers (Med-Associates, Inc., St. Albans, VT, United States) for 1 h daily during their dark cycle. The SA chambers (Med-Associates, Inc., St Albans, VT, United States) had active and inactive levers. An active lever press generated a cocaine infusion (0.5 mg/kg, 0.13 ml, 5 s/infusion; cocaine was obtained from the National Institutes of Drug Abuse, North Bethesda, MD, United States) through the i.v. catheter, which was connected to an infusion pump. Throughout cocaine infusion intervals, a light located above the active lever was lit for 20 s, 15 s beyond the cocaine infusion period, which lasted only 5 s. During the 15-s intervals, active lever presses were recorded; no additional cocaine reinforcement was provided. Presses on the inactive lever did not activate the infusion pump and light. The number of active lever responses, infusions, and inactive lever responses was recorded. Rats were returned to their home cages at the end of the daily session.

#### Extinction Training

Rats received daily i.p. injections of opipramol (12.5 mg/kg) or baclofen (3.2 mg/kg or 0.1 mg/kg), or O/B (Sigma-Aldrich, Darmstadt, Germany) or saline before each extinction test session. Opipramol was administered 15 min before entering the operant chamber and baclofen 30 min before entering it. The difference in administration time was due to differences in pharmacokinetic properties (Di Ciano and Everitt, 2003; Bareli et al., 2021). An opipramol dose of 12.5 mg/kg was chosen since it was the optimal dose still having an impact but not affecting the locomotion behavior (Bareli et al., 2021). After preliminary experiments with two doses of baclofen (3.2 or 0.1 mg/kg) the lower dose of 0.1 mg/kg was chosen since it was shown to be without sedative side effects (data presented in the first part of Results). Rats were then placed in the operant conditioning chambers for 1-h daily sessions, with no cocaine available and only the light cue turning on, upon active lever presses. Active and inactive lever presses were recorded.

To find the effective dose of baclofen, which is high enough to initiate a therapeutic effect and at the same time does not cause side effects, we examined the effect of two doses of baclofen. In the first one we used a dose of 3.2 mg/kg. This dose was chosen based on previous studies reporting that baclofen in doses 3–5 mg/kg effectively reduces cocaine craving in a rat model. Compared to control rats, the baclofen-treated rats did not move in the experimental cages, so this dose caused side effects. Administration of baclofen (0.1 mg/kg) throughout the extinction phase to rats trained for cocaine self-administration did not reduce active lever presses compared to controls. In Addition, providing a boost of baclofen (0.1 mg/kg) before the second reinstatement to rats that did not respond to opipramol resulted in no change in active lever presses compared to controls.

#### Cocaine-Primed Reinstatement

Twenty-four hours after the last opipramol or baclofen or O/B combination or saline treatment, rats received a single injection of cocaine (10 mg/kg; i.p.) and were placed in the operant chambers for 1 h, with no cocaine available. At the conclusion of the session, rats were immediately anesthetized and decapitated for NAc isolation and further molecular analysis (below).

#### Tissue Preparation

Rats were decapitated. Their brains were removed rapidly after reinstatement and placed in a rat brain mold (constructed at Bar Ilan University) on ice. Then, the tissue was dissected into serial 1 mm slices and placed on chilled microscope slides. Tissue punches of the NAc were procured rapidly according to the following coordinates: AP, +1.4 mm; ML, +1.2 mm to bregma (Paxinos and Watson, 2007), using a stainless-steel cannula with an inner diameter of 1.1 mm. The tissue samples were immediately frozen on dry ice and stored at −80°C until they were extracted for analysis.

#### RNA Extraction and Reverse Transcription

We performed analyses of gene expression for Gabbr1 of saline-treated rats (*n* = 8–9) and opipramol-treated rats (*n* = 12) and for Rac1 of O/B treated rats (*n* = 7), also. Total RNA was extracted using a Total RNA Purification Kit (Norgen Biotek Corp., Thorold, Canada). Purity, integrity, and concentration of the isolated RNA samples were determined by spectrophotometric absorbance at 260 nm. RNA samples were reverse transcribed to generate cDNA (qScript cDNA Synthesis; Quanta BioSciences Inc., Gaithersburg, MD, United States). These were later used as templates for quantitative real-time PCR analysis. Real-time PCR reactions were carried out on a Step One Plus Real-time PCR system (Thermo Fisher Scientific, Waltham, MA, United States) using fluorescent SYBR Green fast mix technology (qScript cDNA Synthesis; Quanta BioSciences Inc., Gaithersburg, MD, United States). Reaction protocols were as follows: 30 s at 95°C for enzyme activation followed by 45 cycles of 5 s at 95°C and 30 s at 60°C. Melting curve analysis examined the specificity of the amplification products. Primers [synthesized by Integrated DNA Technologies (IDT), Coralville, IA, United States] for tested genes were designed as follows: Gabbr1 (F:5′-ACGTGGCTTGGCATTTTCTATG-3′; R:5′-TTC AGTGGACACGCTCTTGG-3′), Rac1 (F: 5′-AAAACCAGTGA ATCTGGGCCT-3′; R: 5′-AACACGTCTGTTTGCGGGTA-3′) and beta-actin (F: 5′-GTAGCCATCCAGGCTGTGTT-3′; R: 5′-CCCTCATAGATGGGCACAGT-3′).

## The Rationale of the Study

The study is based on our previous publication (Tsai et al., 2012) in which we showed that treatment with opipramol in a cocaine self- administration (SA) rat model, caused reduction of cocaine-primed reinstatement in 75% of the opipramol-treated group (i.e., Rs). Using a K-mean cluster analysis, the opipramol-treated group was shown in our previous study to constitute two separate groups: Rs and Non-Responders (NR) to opipramol treatment in a cocaine SA model. The separation between the two groups had a specific cutoff point.

In an attempt to augment the efficacy of opipramol we added baclofen (0.1 mg/kg) to opipramol and assessed the rate of rat-R to the combined treatment in comparison to opipramol alone.

The aim of the present study was to assess whether of addition of baclofen to opipramol increases the rate of treatment success achieved by opipramol alone. The focus of this study were rats not affected by opipramol alone [opip (NR)]. To this end, in the first stage we identified the opip (NR) rats as assessed by their craving state in the reinstatement test. Once identified, we gave these rats an acute baclofen injection and retested their craving in a second reinstatement.

In our previous study using the same model, we demonstrated that there is no order effect in the reinstatement tests (Bareli et al., 2021).

## Results

### Baclofen Administered in High Doses of 3.2 mg/kg Induces Sedative Effects and Is Ineffective in Lowering Cocaine Cravings

**FIGURE 1:**
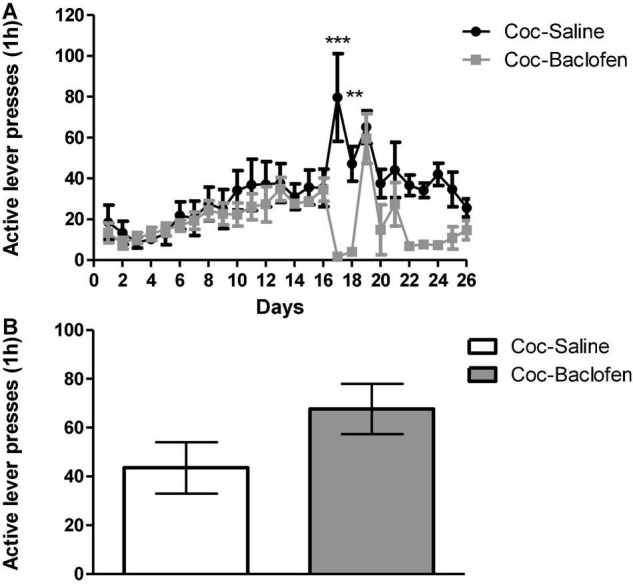
Effects of a dose of 3.2 mg/kg of baclofen on extinction, withdrawal and reinstatement phases in a cocaine (Coc) self-administration (SA) rat model. **(A)** Effect of baclofen on cocaine-seeking during extinction and withdrawal phases. Rats (*n* = 6) were trained for cocaine (0.5 mg/kg) or saline SA on days 1–16. Coc-Saline, control, trained for cocaine SA and received saline at the withdrawal phase, and Coc-Baclofen, trained for cocaine SA and received baclofen (3.2 mg/kg) at the withdrawal phase. Baclofen treatment, given on days 17–18, significantly reduced cocaine-seeking behavior throughout extinction training, compared with saline-treated controls. A two-way ANOVA with repeated measures revealed a significant effect of treatment [*F*(1, 250) = 13.93, *p* = 0.0039], time [*F*(25, 250) = 4.77, *p* < 0.0001], and the interaction between them [*F*(25, 250) = 3.11, *p* < 0.0001] (Bonferroni, ***p < 0.001 day 17, **p < 0.01 day 18). A lack of baclofen administration on day 19 increased active lever presses immediately and resembled control. Baclofen administration on days 20–26 reduced active lever presses but not significantly. **(B)** Effect of Baclofen on cocaine-primed reinstatement. Rats received a priming injection of cocaine (10 mg/kg, i.p, day 27) and were placed in the SA chamber with no cocaine available in the relapse test. Baclofen treatment did not decrease active lever pressing compared to controls (Student’s *t*-test, *p* = 0.1323). Values are expressed as mean ± SEM.

A high-dose of baclofen (3.2 mg/kg) was administered in a classical cocaine SA rat model according to a FR-1 schedule. This dose was chosen based on previous studies reporting that baclofen in doses of 3–5 mg/kg effectively reduces cocaine cravings. The rats (*n* = 6) demonstrated stable cocaine SA behavior during 1-h/day training sessions over 16-days (Figure 1A). At stable maintenance, rats were divided into two groups: one received consecutive injections (i.p.) of baclofen (3.2 mg/kg per day, 30 min before the session) and the other at the same time point, of saline. The saline injected rats served as control group. Both groups went through extinction on days 17–26. The baclofen-treated rats stopped completely active lever presses at the beginning of the extinction phase. Compared to control rats, the baclofen-treated rats showed little movement in the experimental cages, probably due to the sedative effect of the high dose of baclofen. We tested the consequences of not administrating baclofen treatment on the following day (day 19). Active lever presses were dramatically increased to the same level as that of the saline-treated rats. As our aim was to examine whether chronic baclofen treatment attenuates cravings, we continued administration on the consecutive days (days 20–26). Baclofen injections were discontinued on day 26 (last extinction session), and 24 h later, both baclofen-treated and control rats received a priming injection of cocaine (10 mg/kg; i.p.) and were placed in the SA chambers for 1 h. We did not find any differences in active lever presses between baclofen-treated rats and controls (Figure 1B).

### Baclofen Administered at Low Doses of 0.1 mg/kg, as Sole Treatment and in Combination With Opipramol, to Rats That Are NRs to Opipramol Alone, Did Not Reduce Cocaine Cravings

**FIGURE 2:**
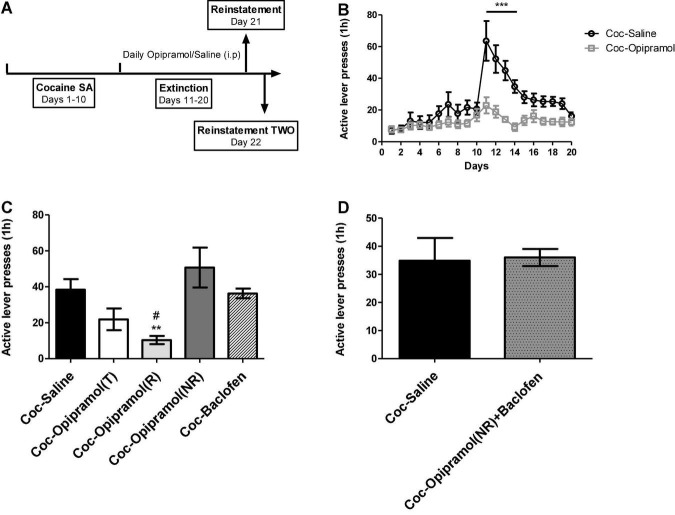
Effect of opipramol (12.5 mg/kg) and low doses of baclofen (0.1 mg/kg) on extinction, withdrawal and reinstatement phases in a cocaine (Coc) self-administration (SA) rat model. **(A)** The fixed ratio 1 schedule (FR-1) of cocaine SA model that was used. Rats (*n* = 10–14) were trained (T) for cocaine (0.5 mg/kg) or saline SA on days 1–10. Next, we created two groups: Coc-Saline, control, trained for cocaine SA and received saline at the withdrawal phase, and Coc-Opipramol, trained for cocaine SA and received opipramol (12.5 mg/kg) throughout the withdrawal phase. **(B)** Effect of opipramol on cocaine-seeking during extinction and withdrawal phases. Opipramol treatment, given on days 11–20, significantly reduced cocaine-seeking behavior during extinction training, compared with saline-treated controls. A two-way ANOVA with repeated measures revealed a significant effect of treatment [*F*(1, 418) = 12.64, *p* = 0.0018], time [*F*(19, 418) = 15.49, *p* < 0.0001] and the interaction between them [*F*(19, 418) = 6.65, *p* < 0.0001] (Bonferroni, ***p < 0.001 days 11–14). **(C)** Effect of opipramol or baclofen on cocaine-primed reinstatement. Classification of treated rats according to “responders” or “non-responders” to chronic opipramol treatment revealed significantly decreased active lever presses for responder rats compared with non-responders or controls (Coc-Saline). A one-way ANOVA revealed a significant effect of treatment [*F*(4, 34) = 5.35, *p* = 0.0019] in the relapse test (Bonferroni, **p < 0.01 opipramol responders (R) vs. non-responders (NR), ^#^*p* < 0.05 opipramol responders vs. controls). Baclofen administration did not decrease active lever presses compared to controls. Coc-Opipramol (T) included the total rats that received opipramol, i.e., opipramol responders [Coc-Opipramol (R)] and non-responders [Coc-Opipramol (NR)]. **(D)** Examination of baclofen (0.1 mg/kg) one injection to opipramol non-responder rats, before a second cocaine-primed reinstatement. A Student’s *t*-test did not reveal a significant treatment effect in the relapse test (*p* = 0.9207). Values are expressed as mean ± SEM.

The reinstatement timeline is presented in Figure 2A. We previously reported that in the SA model of cocaine SA, two groups emerged with regard to chronic opipramol administration: Rs (75%) and NRs (25%) to opipramol (Bareli et al., 2021). Those groups were defined by K- mean cluster analysis as Rs being rats that showed a significantly lower number of active lever presses during reinstatement in contrast to NRs, which did not show such reduction. Among cocaine-trained rats, 75% showed a reduction in cocaine cravings (see Figure 2C), probably resulting from activation of the σ-1 receptor, which is present in the NAc as are Rac1 and Gabbr1. As the baclofen’s target is the Gabbr1, we postulated that the O/B combination may increase the number Rs, compared to opipramol alone. As described earlier, rats that had been trained for cocaine SA were divided into three groups: one group received injections (i.p.) of opipramol (12.5 mg/kg per day, 15 min before the session), the second group received injections (i.p.) of baclofen (0.1 mg/kg per day, 30 min before the session) and the third group received saline (30 min before the session) as a control for the baclofen. Next all groups went through an extinction test session (on days 11–20). Active lever presses decreased significantly in the opipramol-treated rats, compared to the saline-treated group, throughout the extinction phase (Figure 2B). Administration, throughout the extinction phase, to rats trained for cocaine SA of baclofen (0.1 mg/kg; Coc-Baclofen) did not reduce active lever presses compared to controls (Coc-Saline; Figure 2C). In Addition, providing a boost of baclofen (0.1 mg/kg) before the second reinstatement to rats that were NRs to opipramol (Coc-Opipramol (NRs)) resulted in no change in active lever presses compared to controls (Figure 2D).

### Chronic Treatment With a Combination of Opipramol and a Low Dose of Baclofen During the Extinction Phase Is More Effective in Reducing Cocaine Craving Than Opipramol Alone

**FIGURE 3:**
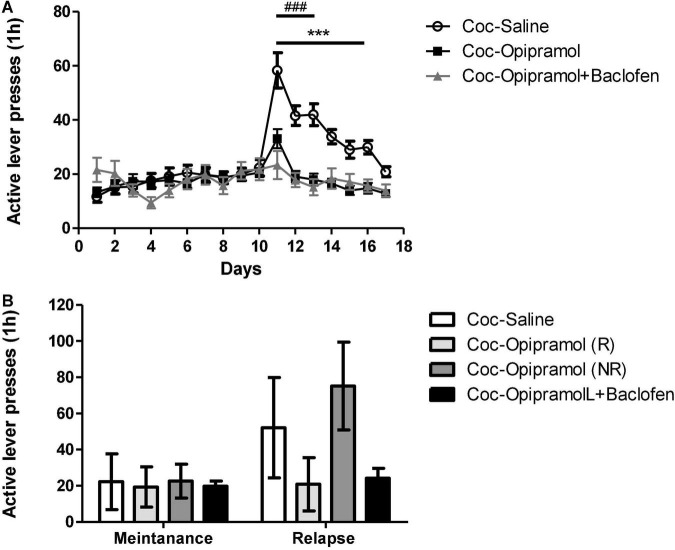
Effect of the combination of opipramol and baclofen on extinction, withdrawal and reinstatement phases in a cocaine (Coc) self-administration rat (SA) model. **(A)** Effect of the selected treatment; opipramol (12.5 mg/kg) or opipramol (12.5 mg/kg) and baclofen (0.1 mg/kg) combination on cocaine-seeking during extinction and withdrawal phases. Opipramol alone and the combination treatments given on days 11–17 significantly reduced cocaine-seeking behavior during extinction training, compared with saline-treated controls. A two-way ANOVA with repeated measures revealed a significant effect of treatment [*F*(2, 1712) = 7.42, *p* = 0.001], time [*F*(16, 1712) = 16.97, *p* < 0.0001] and the interaction between them [*F*(32, 1712) = 7.75, *p* < 0.0001]. (Bonferroni, ***p < 0.001 days 11–16 opipramol treatment vs. controls, ^###^*p* < 0.001 days 11–13 O/B treatment vs. controls). **(B)** Effect of opipramol or the combination of O/B on cocaine-primed reinstatement. Active lever presses were decreased in rats that were responders (R) to opipramol [Coc-Opipramol (R)] and in rats that received O/B combination (Coc-Opipramol + Baclofen) compared with opipramol non-responder (NR) rats [Coc-Opipramol (NR)] and controls (Coc-Saline). A one-way ANOVA revealed a significant effect of treatment [*F*(3, 89) = 28.97, *p* < 0.0001] in the relapse test (Bonferroni, ***^/###^*p* < 0.001 opipramol responders and O/B vs. non-responders and controls, respectively). Values are expressed as mean ± SEM.

The above-described results and the narrow therapeutic index of baclofen led us to examine whether chronic administration of a combination of opipramol and a low dose of baclofen (O/B) throughout the extinction phase would increase the number of rats responding to opipramol. The rats were put through the same cocaine SA paradigm as described above. Active lever presses decreased significantly in the O/B-treated rats compared to controls (Figure 3A). Furthermore, in the relapse test, the combination treatment reduced active lever presses and increased the rate of Rs compared to R-rate to opipramol alone (Figure 3B).

### Combination of Opipramol and Baclofen Changes Expression Levels of Proteins in the Nucleus Accumbens

In a previous study we showed that opipramol alone acts on a complex containing σ-1r and Rac1 proteins (Bareli et al., 2021). The present study found that craving behavior was affected by administration of O/B to cocaine-trained rats.

**FIGURE 4:**
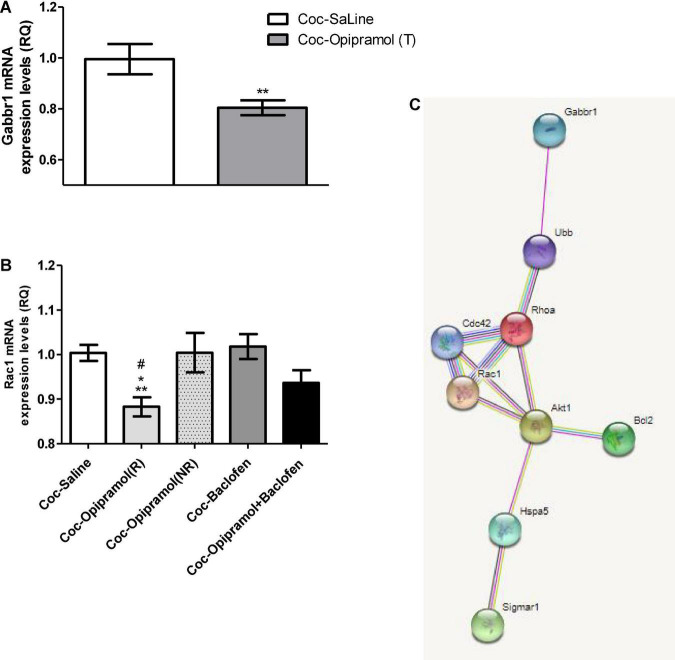
Effects of the various drug-combinations on Gabbr1 and Rac1 mRNA expression levels in the nucleus accumbens (NAc). **(A)** A Student’s *t*-test revealed a significant effect of opipramol treatment alone on the expression of Gabbr1 (**p = 0.0063). **(B)** Newman-Keuls *post hoc* test revealed a significant decrease in Rac1 mRNA expression levels in the opipramol responders (R) [Coc-Opipramol (R)] compared with the non-responder (NR) rats [Coc-Opipramol (NR)], as well as compared to high dose baclofen (3.2 mg/kg) alone treated rats (Coc-Baclofen), and compared to controls (Coc-Saine) (one-way ANOVA, *p < 0.05, ^#^*p* < 0.05, and **p < 0.01, respectively). Values are expressed as mean ± SEM. **(C)** STRING software (version 11.0) revealed a complex of interacting proteins including Gabbr1, Rac1, and σ-1 receptor in a PPI enrichment analysis, *p* = 0.0272. The software indicates that the examined proteins are at least partially biologically connected.

Baclofen’s main target is Gabbr1 thus we examined Gabbr1 involvement in the beneficial effect of the treatment. qPCR analysis revealed a significant decrease in the mRNA expression levels of Gabbr1 in the opipramol treated group compared to controls (^**^p < 0.01, Figure 4A). High doses of baclofen (3.2 mg/kg) did not affect Rac1 mRNA expression levels (Figure 4B). Administering the combination of opipramol (12.5 mg/kg) with a low dose of baclofen (0.1 mg/kg) did not lead to a significant decrease in Rac1 mRNA expression levels (Figure 4B). According to the STRING analysis, σ-1r, Rac1, and Gabbr1 all the examined proteins interact (Figure 4C).

## Study 2—Human Trial

### Materials and Methods

#### Clinical Trial Design

**FIGURE 5:**
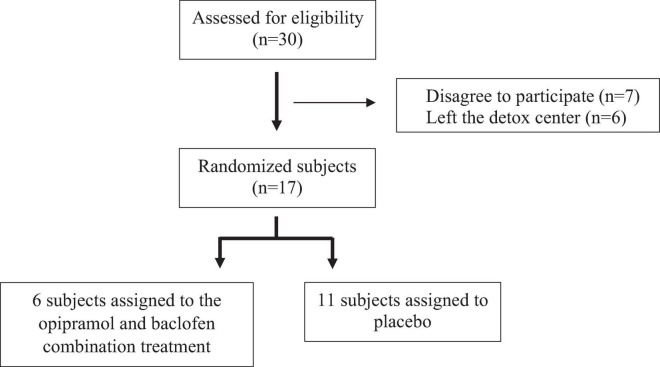
Subject recruitment. Participants were randomly assigned to either the opipramol-baclofen (O/B) combination group or to the placebo group.

**FIGURE 6:**
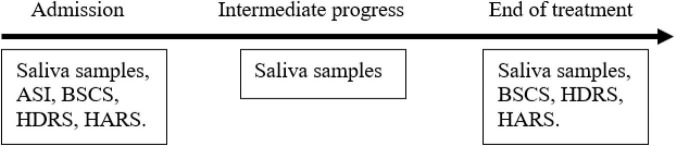
Study timeline. The clinical scales and saliva samples were collected on days 2 and 7 of the trial and 1 day before the release from the detox center. ASI, Addiction Severity Index; BSCS, Brief Substance Craving Scale; HDRS, Hamilton Depression Rating Scale; HARS, Hamilton Anxiety Rating Scale.

Residential detoxification centers provide a controlled environment that enables a balanced diet and a fairly stable daily routine, thus providing a controlled research setting. We designed a 1-month study during which the patients were administered either O/B treatment or placebo (Figure 5). Behavioral, clinical, and hormonal/biochemical parameters were monitored (Figure 6). All adverse effects, spontaneously reported by the study participants, were assessed for severity and association with study medication.

#### Ethics

The study was approved by the review board of the Israeli Ministry of Health (proposal no. 066-2015, Aug 2017). All participants signed a written informed consent form.

#### Participants

Thirty people with PsUD from a residential detoxification center (Dolphine, Ashdod, Israel) participated in the study. Inclusion criteria were a diagnosis of PsUD, age in the range of 18–60 years. Exclusion criteria consisted of the following: serious kidney, lung, liver, neurological, prostatic or cardiovascular disease, presence of suicidal thoughts or behaviors (as assessed in a clinical interview), acute or chronic psychosis, bipolar disorder, major depressive episodes, intellectual disability, organic brain syndrome, current treatment with monoamine oxidase (MAO) inhibitors or benzodiazepines, being pregnant or breast-feeding and finally, active HIV or hepatitis C. Formal cognitive assessments were not conducted in this study. However, all participants underwent a clinical interview at baseline that included assessment of their basic cognitive skills. Those found to have gross cognitive impairment were also excluded from the study.

#### Protocol and Medication Procedure

Participants were randomly assigned to either the O/B combination group or to the placebo group. Each participant was assigned a random number generated by a random number generator, thus keeping the participants’ identity unknown to the researchers (Matthews, 2003). O/B or placebo were prepared by Nextar Chempharma Solutions Ltd. (Rehovot, Israel) and were administered by a registered nurse. For the duration of 1 month, those assigned to the study group received 30 mg of baclofen and 50 mg of opipramol that were given daily by oral administration in 3 divided doses over the day. All patients were asked at baseline, to stop using drugs or medications that affect cravings (such as benzodiazepines, antidepressants, metadoxine, naltrexon, acamprosate, γ-hydroxybutyric acid, antihistamines) for the duration of the study. Each patient was monitored throughout the study period (Figure 6). Professional staff provided a standard program for the treatment of SUDs, including relapse prevention, 12 steps, motivational enhancement and daily group interventions. At specific time points, patients completed a battery of scales measuring cravings for addictive substances (Addiction Severity Index—ASI; Brief Substance Craving Scale—BSCS), anxiety [Hamilton Anxiety Rating Scale—HARS)] and depression [Hamilton Depression Rating Scale—HDRS)]. Saliva samples were also collected in order to evaluate DHEA-S and cortisol hormone levels (Figure 6). Throughout the detoxification period all participants received group psychotherapy along with the pharmacotherapy.

#### Assessments

Testing started in the first 2 days of a patient’s admission to the detox center. Data recorded at baseline included standard demographics consisting of sex, age, marital status, immigration status, date of immigration, and education level. Additionally, each patient’s history of psychiatric disorders was recorded, as well as the patient’s SUD history. The following details were collected for each participant: (1) age at first substance use, (2) age at first binge, (3) duration of substance consumption, (4) number of prior detoxification programs (inpatient and outpatient), (5) average daily substance consumption in the last 6 months, (6) the number of days in the last month when substances were consumed.

Patients were administered the following measurement tools: (1) ASI, a semi-structured interview designed to address the following 7 potential problem areas in patients with SUD: medical status, source of income, drug use, alcohol use, legal status, family/social status, and psychiatric status. The ASI provides a general overview of problems related to substance use rather than focusing on any single problem area. (2) BSCS, a self-report instrument that assesses cravings for multiple substances with regard to: intensity and frequency of cravings, number of times per week, thoughts of cravings for a substance). (3) HDRS, a scale frequently used by clinicians to assess depressive symptoms. (4) HARS, a scale used by clinicians to assess anxiety symptoms. In our analysis we calculated three parameters related to SUD—depression, helplessness, and worthlessness feelings from Hamilton Depression Rating Scale (HDRS) (Yadid et al., 2012; Barnett et al., 2018).

As shown in Figure 6 below, saliva samples were taken from the participants three times during the study: at the beginning of the study, in its middle and at the end. At the beginning and the end of the study along with the saliva samples that were taken for hormone analysis scales for clinical assessments were completed as well.

#### Analysis of Saliva Samples

Saliva samples were taken before breakfast, between 06:30 and 07:30 a.m., and were used to analyze levels of DHEA-S and cortisol. The latter were measured with DHEA-S/Cortisol Saliva ELISA kits (IBL International GmbH, Hamburg, Germany). The sensitivity of DHEA-S and cortisol kits are 0.05 ng/ml and 0.005 μg/dl, respectively, and both present low cross-reactivity to other hormones.

#### Data Analysis

All data are expressed as mean ± SD. One-way ANOVA or two-way ANOVA with repeated measures (days) followed by Bonferroni or Student-Newman-Keuls *post hoc* test were used as appropriate. A Student’s *t*-test was used for comparisons between the two groups. All the data were analyzed using Prism 5 software (GraphPad, San Diego, CA, United States).

### Results

#### Opipramol and Baclofen Combination Treatment Improves Depressive Symptoms, Craving and Stress Hormones in People With Polysubstance Use Disorder

Following the promising results from the rat model study, we performed a double-blind placebo-controlled pilot study to investigate whether the O/B combination can lower addiction and anxiety/depressive symptoms. The demographic and clinical characteristics of the study population are presented in Table 1.

**TABLE 1:** Demographic and clinical characteristics of the study population.

	Placebo (*n* = 11)	O/B treatment (*n* = 6)
Age (years; mean ± SD)	40 ± 10.5	40.5 ± 10.8
Male: Female	5:1	9:2
Stimulants	6	7
Opioids	5	11
Depressants	3	6
Hallucinogens	4	6

*O/B, Opipramol and baclofen.*

**FIGURE 7:**
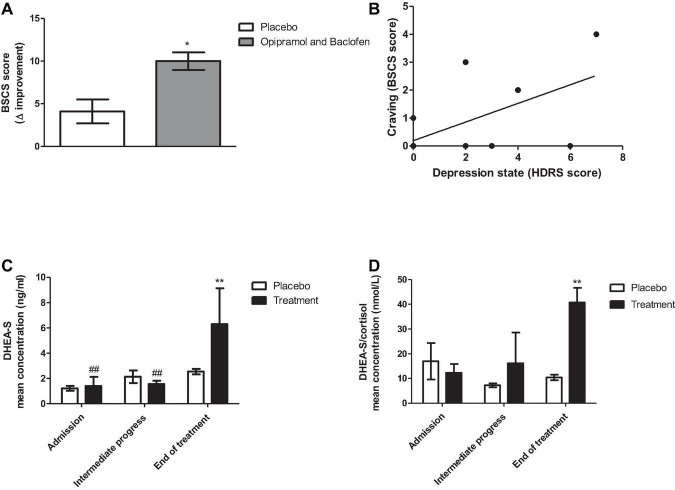
Effects of administering a combination of opipramol and baclofen on rehabilitation factors of patients with polysubstance use disorder (PsUD). **(A)** The individual magnitude of craving change tested by the Brief Substance Craving Scale (BSCS) questionnaire (score before/after O/B treatment was significantly improved in comparison with placebo treatment (Student’s *t*-test, *p* < 0.031). **(B)** A correlation between depression state and craving symptoms at the end of the trial (Pearson rank correlation, *p = 0.022, *r* = 0.36). **(C)** Dehydroepiandrosterone- sulfate (DHEA-S) levels were increased at the end of the treatment compared with placebo (Bonferroni, **p < 0.01) and over time (Bonferroni, ^##^*p* < 0.01, admission vs. end of treatment, intermediate progress vs. end of treatment). A two-way ANOVA with repeated measures revealed a significant effect of time [*F*(2, 20) = 12.85, *p* = 0.0003] and interaction between treatment and time [*F*(2, 20) = 6.22, *p* = 0.0079], but did not reveal a significant effect of treatment [*F*(1, 20) = 2.27, *p* = 0.1628]. **(D)** DHEA-S and cortisol ratio were increased at the end of the treatment compared with placebo (Bonferroni, **p < 0.01). A two-way ANOVA with repeated measures revealed a significant effect of treatment [*F*(1, 16) = 9.2, *p* = 0.0162 and interaction between treatment and time [*F*(2, 16) = 3.67, *p* = 0.0488], but did not reveal a significant effect of time [*F*(2, 16) = 2.52, *p* = 0.1119].

The outcomes of the treatments are shown in Figure 7. Patients with PsUD receiving the treatment combination three times a day, demonstrated higher levels of improvement in cravings compared to placebo. Moreover, we observed a significant correlation between depression state and craving. Remarkably, at the end of the study DHEA-S levels were higher in the study group than in the placebo group. The ratio of DHEA-S/cortisol was also high throughout the trial.

### Discussion

The current study shows that administration of the O/B combination in humans with PsUD, lowers cocaine cravings and possibly the risk of relapse and reduces the depressive symptoms and increases at the end-point, the level DHEA-S. In the rat model, the addition of baclofen to opipramol resulted in a significant reduction in the relapse to cocaine rate of rats that did not respond to opipramol.

On the molecular level, our findings suggest that the beneficial effect of O/B may be mediated by the interaction of proteins, including GABA_*B*_ receptors, σ-1 receptor, and Rac1.

Our previous study Bareli et al. (2021) showed that Rac1 has a critical role in drug-seeking and cravings. We also showed that opipramol significantly decreased σ-1 receptor mRNA levels, indicating a correlation between Rac1 and σ-1 receptor when responding to opipramol. Both σ-1 and GABA_*B*_ receptors are proteins that interact during changes in cellular function through crosstalk with other proteins, regulating signal transduction, receptor localization, and pharmacological profiles (Hayashi and Su, 2007; Nguyen et al., 2015; Terunuma, 2018). Changes in the GABA_*B*_ receptor functions have been linked with an array of psychiatric disorders, including depression, anxiety, cognition, nociception, and SUD (Möhler, 2012; Felice et al., 2016; Terunuma, 2018). Gabbr1 associates with the σ-1 receptor, as demonstrated by increased expression of Gabbr1 in the CA1 region of the hippocampus in σ-1 receptor-knockout-mice (Vavers et al., 2021) and, as shown in the current study, changes in Gabbr1 and in Rac1 in the NAc of rats treated with opipramol.

Animal studies revealed that the efficacy of baclofen depends on the dose selected, both for cocaine and baclofen, and the setup, i.e., the requirement of a FR schedule rather than a progressive ratio schedule. Campbell et al. (1999) showed that high-doses of baclofen (2.5, 5 mg/kg) decrease SA of low doses of cocaine and attenuate cravings (Campbell et al., 1999). Their addiction model, however, was not a longitudinal one, testing the effect on substance withdrawal. Another study, using 5 mg/kg of baclofen for 5 days under FR-5, significantly decreased cocaine SA (Shoaib et al., 1998). In contrast, Roberts et al. (1996) demonstrated that a dose of 1.5 mg/kg baclofen did not reduce cocaine SA at an FR-1 schedule but had a positive effect on a progressive ratio schedule that emphasizes the stressful/obsessive manner of substance consumption (Roberts et al., 1996). Eleven randomized clinical trials that tested baclofen efficacy in patients with withdrawal syndrome or SUDs reported inconsistent results. About half of those studies reported baclofen as being effective (Agabio et al., 2013), whereas others showed minor or no differences between baclofen and placebo (Ahmadi-Abhari et al., 2001; Heinzerling et al., 2006; Garbutt et al., 2010; Bschor et al., 2018).

In our study, we attempted to find an optimal dose of opipramol in the O/B combination, for the treatment of SUD. To the best of our knowledge, opipramol was never tested in pre-clinical trials in relation to SUD. We used a rat model in order to identify the dose that elicits a cocaine-craving inhibiting effect without affecting locomotion (Bareli et al., 2021).

In clinical trials, it is common to administer a target dose of 200 mg/kg opipramol a day for treating generalized anxiety and somatoform disorders (Volz et al., 2000; Möller et al., 2001). Most clinical trials use a maintenance dose of 30–300 mg baclofen a day (de Beaurepaire et al., 2019).

In our animal model for cocaine cravings, the dose of baclofen administered to the rats was reduced to a minimal one. We thus decided that in order to achieve a clinical effect with minimal side effects when combining opipramol and baclofen, which share a common molecular target, a dose of 150 mg/kg opipramol, and 90 mg/kg baclofen a day, may help inhibit cocaine cravings.

SUDs often co-occur with mood disorders. The reasons vary from genetic tendencies (Quello et al., 2005) to using substances as self-medication in an attempt to relieve depression (Markou et al., 1998). It is often difficult to differentiate between pure mood disorders and SUD associated with or mimicking the symptoms of mood disorders. In either case it is essential to manage the depression that is associated with SUD. In the past treatment was directed at either SUD or depression (Westermeyer, 2003), however, it was shown that both components have to be addressed simultaneously (Pettinati, 2004).

Treatments for SIDD include mood- stabilizers for withdrawal symptoms and antidepressants for mood symptoms (Nunes and Levin, 2004). In clinical trials, using antidepressants, especially selective serotonin reuptake inhibitors (SSRIs), resulted in unclear outcomes (Nunes and Levin, 2004; Pettinati et al., 2013; Agabio et al., 2018). A large multi-center randomized, single-blind placebo trial, conducted in patients with co-occurring major depressive disorder and alcohol dependence, showed that administration of the SSRI sertraline did not improve depression or drinking behavior, compared to placebo (Kranzler et al., 2006). However, the simultaneous administration of two medications, sertraline for depression and naltrexone for alcohol dependence, to patients with depression co-morbid with alcohol dependence showed an advantage in decreasing symptoms of both disorders (Pettinati et al., 2010). In another double-blind, placebo-controlled trial, patients were randomly assigned to four groups receiving 14 weeks of treatment with sertraline, naltrexone, or sertraline/naltrexone combination, vs. placebo. Patients treated with the combination exhibited higher rates of alcohol abstinence, their time to relapse to heavy drinking was longer and they tended to have fewer depression symptoms by the end of the treatment (Pettinati et al., 2010). This study supports our results with regard to the beneficial effects of combining two medications, like opipramol and baclofen to treat co-occurring mood disorders cocaine cravings.

The findings of this study indicate that patients receiving daily treatment of combined O/B exhibited improvement in craving symptoms compared to placebo. In addition, mood state was correlated with craving improvement (Figure 7), suggesting a role of mood alterations in the rehabilitation process. We also found at the end of the study, an increase in DHEA-S concentration levels and a significantly higher ratio of DHEA-S/cortisol in patients who received the O/B combination, compared to those who received placebo. This outcome is consistent with studies showing that the ratio between those hormones indicates the levels of anxiety (Ohana et al., 2016).

#### Limitations

While the results verify proof of concept to the behavioral effect in treatment with O/B, there are also limitations. The small sample size resulting from the difficulties recruiting patients and the level of attrition during the study are some of those limitations. Additionally, it is unclear if the short-term effects of our treatment are maintained long-term.

All the participants received group psychotherapy interventions along with pharmacotherapy, throughout the detoxification period. Thus, some of our outcomes may have been affected by the psychotherapy, however, both study subjects and controls received the same psychotherapy.

The O/B combination may be a promising approach for SUD, but this combination may produce more secondary effects than using each medication alone (Khezrian et al., 2020). Future studies should evaluate the beneficial effect of each one of these drugs alone in comparison to the combination, in a similar population.

## Conclusion

Our preliminary clinical trial results may encourage researchers to conduct a long-term, double blind, placebo controlled, multi-center clinical trial to examine the effectiveness of the O/B combination as a novel anti-anxiety, anti-depression and anti-craving treatment on a large sample of patients with PsUD.

## Data Availability Statement

The original contributions presented in the study are included in the article/supplementary material, further inquiries can be directed to the corresponding author/s.

## Ethics Statement

The study was approved by the Israeli Ministry of Health (proposal no. 066-2015, Aug 2017). The patients/participants provided their written informed consent to participate in this study. The animal study was reviewed and approved by Animal Care and Use Committee of Bar Ilan University, Ramat Gan, and were performed in accordance with the guidelines for animal experiments of the National Institutes of Health.

## Author Contributions

TB contributed to behavioral, molecular, and data analyses, wrote the first draft of the manuscript, conducted the clinical trial, and performed the questionnaires and hormones analysis. HA and RB assisted with the behavioral experiments. HB contributed to the initial behavioral experiments and the former concept of the manuscript. GW assisted with the molecular analysis. IG contributed to the surgical procedures and animal care and rehabilitation. RM drafted the manuscript. PR conceived of the clinical trial and provided the clinical setting for the trial. AW provided a significant revision of the article and prepared the final version of the manuscript. GY contributed to the research concept and design, interpretation of the results, and critical revision of the manuscript. All authors reviewed the content and approved the final version for publication.

## Conflict of Interest

The authors declare that the research was conducted in the absence of any commercial or financial relationships that could be construed as a potential conflict of interest.

## Publisher’s Note

All claims expressed in this article are solely those of the authors and do not necessarily represent those of their affiliated organizations, or those of the publisher, the editors and the reviewers. Any product that may be evaluated in this article, or claim that may be made by its manufacturer, is not guaranteed or endorsed by the publisher.
