# Erratum: Novel Opipramol-Baclofen Combination Alleviates Depression and Craving and Facilitates Recovery From Substance Use Disorder—An Animal Model and a Human Study

**DOI:** 10.3389/fnbeh.2022.952004

**Published:** 2022-06-10

**Authors:** Frontiers Production Office

**Affiliations:** Frontiers Media SA, Lausanne, Switzerland

**Keywords:** stress, substance-induced depressive disorder, addiction, treatment, therapeutic center, baclofen, opipramol

Due to a production error, an author name was incorrectly spelled as Hilla Ben Moshe. The correct spelling is Hila Ben Moshe.

The publisher apologizes for this mistake. The original version of this article has been updated.

